# Identification of CELF1 RNA targets by CLIP-seq in human HeLa cells

**DOI:** 10.1016/j.gdata.2016.04.009

**Published:** 2016-04-19

**Authors:** Olivier Le Tonquèze, Bernhard Gschloessl, Vincent Legagneux, Luc Paillard, Yann Audic

**Affiliations:** a Centre National de la Recherche Scientifique (CNRS), Institut de Génétique et Développement, UMR6290, Rennes, France; b Université de Rennes 1, Rennes 35043, France

**Keywords:** CUGBP1, RNA binding proteins, CLIP-seq, *Homo sapiens*, Alternative splicing

## Abstract

The specific interactions between RNA-binding proteins and their target RNAs are an essential level to control gene expression. By combining ultra-violet cross-linking and immunoprecipitation (CLIP) and massive SoliD sequencing we identified the RNAs bound by the RNA-binding protein CELF1, in human HeLa cells. The CELF1 binding sites deduced from the sequence data allow characterizing specific features of CELF1-RNA association. We present therefore the first map of CELF1 binding sites in human cells.

## Highlights

• CLIPSeq identifies CELF1 binding sites in human cells.
• CELF1 binds to protein coding, lincRNA and antisense RNA.

## 1 Direct link to deposited data

http://www.ebi.ac.uk/ena/data/view/PRJEB12208.

## 2 Introduction

Regulatory RNA binding proteins play a role in the processing of the RNA molecules by controlling the many steps that follow transcription. This includes but is not limited to nuclear splicing, cleavage and polyadenylation, nuclear export and cytoplasmic localization of the mRNA, cytoplasmic deadenylation, RNA degradation and translational control of messenger RNA.

CELF1 (CUGBP, Elav-like family member 1, also named CUGBP1) is a conserved RNA binding protein that controls alternative splicing in the nucleus and cytoplasmic deadenylation, mRNA stability and translation in the cytoplasm [5]. It has been implicated in several pathological conditions. CELF1 is over-expressed in Myotonic Dystrophy, type I (DM1), and several animal models revealed that this overexpression is an important trigger of DM1 symptoms [2]. CELF1 was also found to be deregulated in several human cancers, and may contribute to cell transformation [12], [19], [31]. Finally, genome-wide studies revealed an association between CELF1 locus and Alzheimer disease [15], [17] suggesting putative relationships between CELF1 and neurodegeneration.

These findings highlight the need for the systematic identification of the repertoire of RNAs associated with CELF1 in human cells. Analysis of known CELF1 RNA ligands and of *in-vitro* selection experiments identified UGU-rich motifs as required for CELF1 binding [14], [18], [20], [24], [26]. However, this information is not discriminative enough to allow for the identification of CELF1 binding sites in transcripts solely based on their sequence. A widely used method for mapping protein-RNA interactions *in-vivo* relies on UV cross-linking and immunoprecipitation (CLIP) of RNA-binding proteins [29] followed by deep sequencing (seq) of the co-purified RNAs. We describe here the results of CLIP-seq experiments to identify the RNA binding sites of CELF1 in human HeLa cells.

## 3 Results and discussion

### 3.1 CLIP-seq

**Fig. 1:**
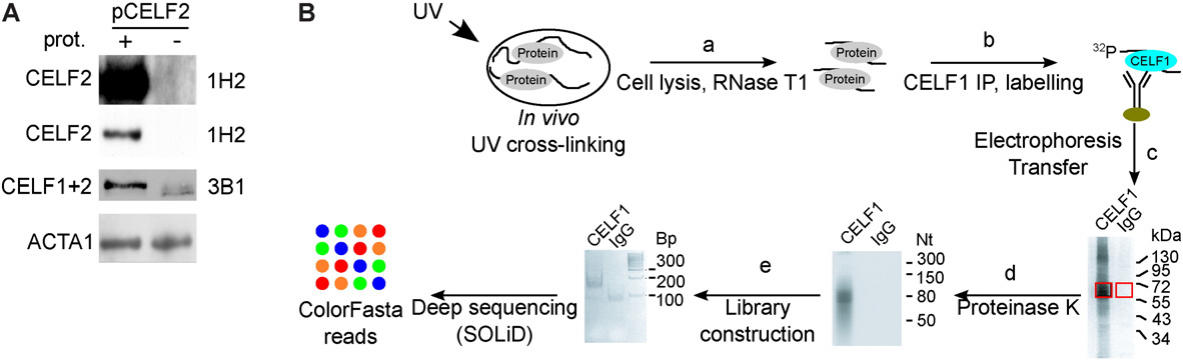
CELF1 CLIP-seq A) Western blots of HeLa cells transfected with a *CELF2* expression plasmid (+) or a mock plasmid (−). The antibodies reveal CELF1 + 2 (3B1), CELF2 (1H2) or ACTA1. B) Biochemical protocol of CLIP and library preparation.

CELF1 (CUGBP, Elav-like family member 1, also named CUGBP1) is a founding member of the CELF family of RNA-binding proteins [1]. Among the CELF members, CELF2 is the closest paralogue of CELF1, with which it shares extensive sequence conservation. We were therefore concerned about the possibility that the anti-CELF1 monoclonal antibody used in our work (3B1) could react with CELF2. Indeed, the signal obtained in a Western experiment with this antibody was reinforced in cells transfected with an expression vector directing a strong expression of *CELF2* (Fig. 1A), indicating that, in our hands, the 3B1 antibody recognizes CELF2. However, we detected no signal with an antibody against CELF2 (1H2) in HeLa cells, even on an overexposed blot, unless the cells were transfected with the CELF2 expression vector (Fig. 1A, upper panel), showing that CELF2 is not expressed in these cells. This reveals that only RNAs associated with CELF1 can be retrieved from CLIP experiments run in these cells.

The CLIP-seq protocol is presented in Fig. 1B. HeLa cells grown at 30% confluence were UV-irradiated (254 nm) to create covalent bonds between nucleic acids and associated proteins *in-vivo*. Cells were rapidly collected and lysed. The cell extracts were treated with RNAse T1 to generate small ribonucleic complexes (Fig. 1B(a)) that were specifically immunoprecipitated using magnetic beads coupled to anti-CELF1 antibody. The immunoprecipitated RNA/protein complexes were radio-labeled with ^32^P-ATP and T4 Polynucleotide Kinase (Fig. 1B(b)). The RNA/protein complexes were depleted of free RNAs by electrophoresis on a polyacrylamide gel and transfer to nitrocellulose (Fig. 1B(c)). The CELF1/RNA complexes were size-selected and proteins were digested by Proteinase K (Fig. 1B(d)). The RNAs isolated from these complexes were used as templates to generate a stranded library by using the Small RNA expression Kit (SREK, SoliD) (Fig. 1B(e)) for SoliD sequencing.

### 3.2 Sequence data and treatment

Two different libraries of CELF1 CLIP-seq (libA and libB) were sequenced in 3 different runs. As a reference, an mRNA-seq library was generated following a protocol as similar as possible to the CLIP library protocol. Briefly, oligo(dT)-selected RNAs were fragmented with RNAse T1, phosphorylated on the 5′end with T4 PNK and a library prepared with the SREK (SoliD). This mRNA-seq library was sequenced in one run.

**Fig. 2:**
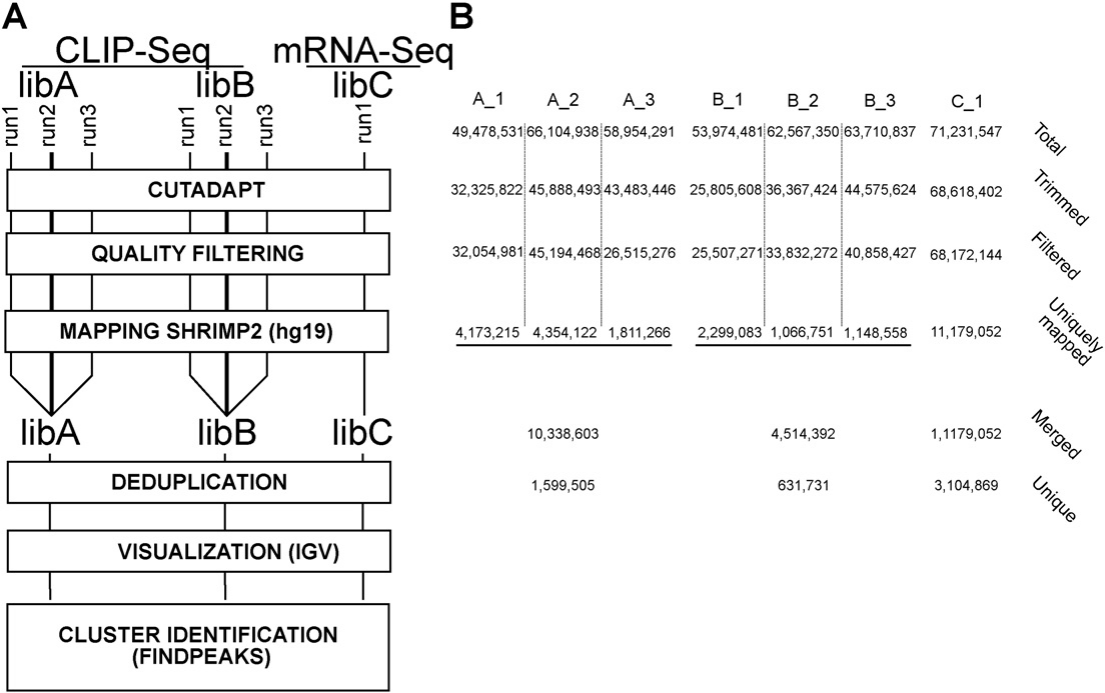
CLIP-seq data A) Treatment of CLIP-seq data for the identification of CELF1 binding sites. B) Table summarizing the number of reads obtained with the CLIP-seq and the mRNA-seq libraries. “Total” is the total number of reads, “Trimmed” and “Filtered” the number of reads after adapter removal and quality filtering, respectively. “Uniquely mapped” is the number of uniquely mapped reads to the human genome. “Merged” is the sum of the uniquely mapped reads arising from independent sequencing runs of the same library. “Unique” is the number of reads remaining after removal of strictly identical reads.

The sequential processing steps are depicted in Fig. 2A. A total of 173 million reads were obtained for libA, 180 million for libB and 71 million for the mRNA-seq (libC) (Fig. 2B). SREK adapters were removed with cutadapt [21] and reads with uncalled bases in the first 30 nucleotides of the sequence were discarded. The reads were then mapped to the hg19 human genome with SHRIMP2 [25]. Only uniquely mapped reads were collected. We obtained 10 million of uniquely mapped reads for libA, 4.5 million for libB and 11 million for the mRNA-seq (Fig. 2B). Mapped reads from each sequencing run were grouped as one file per library. Because read duplications may arise from PCR during library preparation, we chose to keep only one copy of each duplicated read per library. Therefore, cluster identification was performed on unique reads that were uniquely mapped and corresponded to 1.6 millions reads for libA, 0.6 million reads for libB and 3.1 million reads for the mRNA-seq (Fig. 2B). Mapped reads visualization was done on IGV [27], see Fig. 4). We next defined clusters as the genomic regions covered by at least three overlapping reads, and the height of each cluster as the highest number of overlapping reads at a given genomic position within the cluster. A differential analysis using FindPeaks (V4.0, [10]) and the R script “FindPeaksAnalysis.R”, identified the clusters of reads higher in either CLIP-seq library, compared with the mRNA-seq library.

**Fig. 3:**
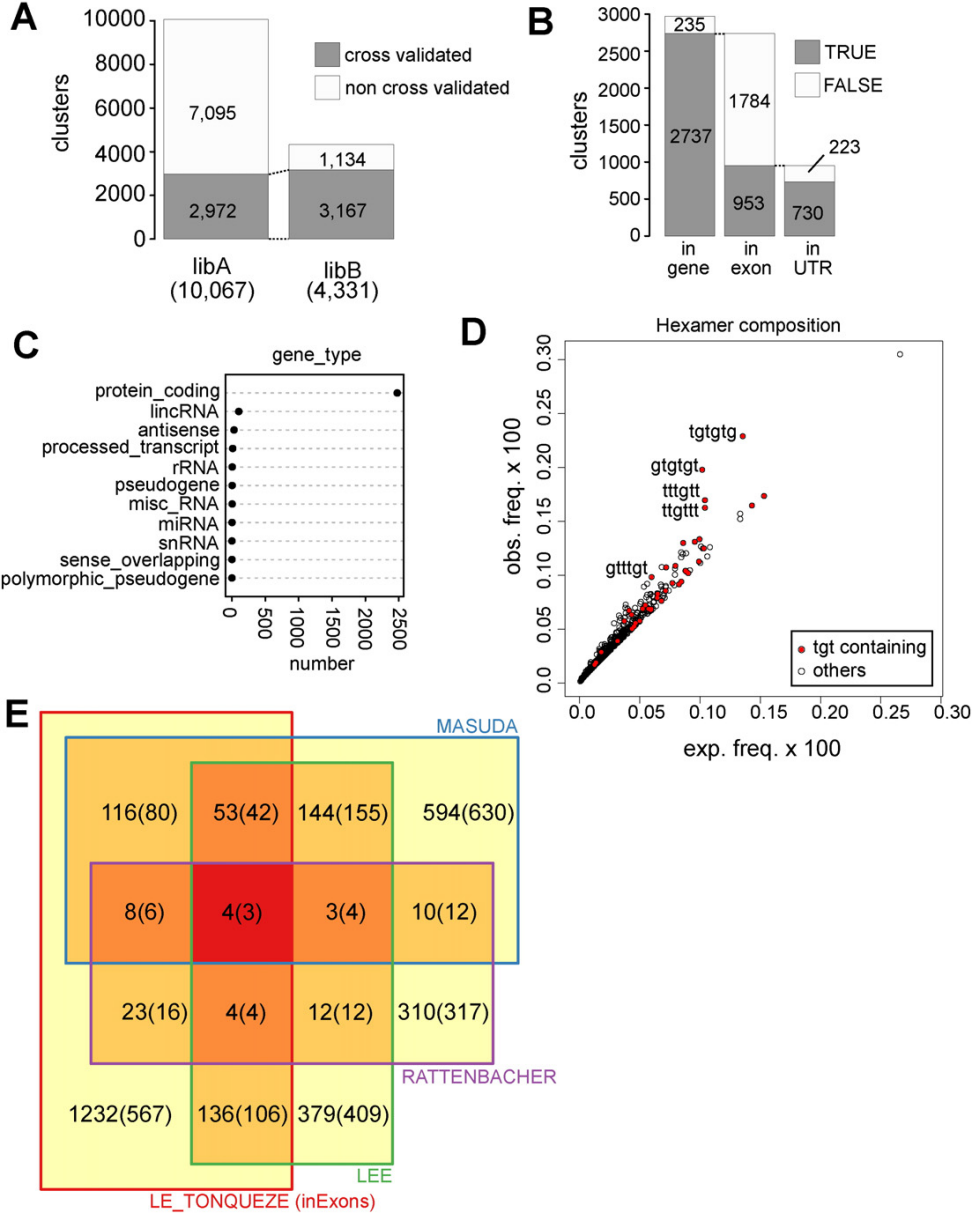
CELF1 binding clusters A) A differential analysis using mRNA-seq data as a reference identified 10,067 and 4331 CELF-binding clusters from libA and libB sequencing data, respectively. We classified a cluster from a library as “cross-validated” when it overlaps a cluster identified in the other library by at least one nucleotide. We show here the number of cross-validated and non cross-validated clusters for each library. B) For the 2972 libA clusters cross-validated by libB, we show the number of genic clusters, then the distribution between exonic and intronic clusters for genic clusters, then the number of exonic clusters in untranslated regions. C) For the 2737 libA genic clusters cross-validated by libB, we show the type of gene. D) For the 2972 libA clusters, we plot the observed frequency of each possible hexamer against its expected frequency calculated from the real frequencies of each dimer. The TGT-containing clusters are in red, and the sequences of the TGT-containing enriched are shown. E) Unweighted Venn diagram illustrating the overlap between CELF1 target genes identified in this work and in [18], [22], [24] based on Ensembl gene ID. Number between parentheses correspond to the comparison with gene harboring CELF1 binding clusters in exonic regions in our analysis.

Respectively, 10,067 and 4331 clusters were considered significantly higher in libA and libB (Supplementary Table 1, Supplementary Table 2). These numbers are consistent with the deeper sequencing of libA (see Fig. 2B). Importantly, about three fourths (3167/4331) of the libB clusters overlap clusters identified in libA (Fig. 3A**,** Supplementary Table 2), demonstrating the reproducibility of the CLIP replicates. We next focused on the 2972 clusters found in libA with a cross-validation by libB (Supplementary Table 1), which we termed CELF1-binding clusters. We annotated them based on the Gencode annotation (Release 19 (GRCh37.p13)). More than 90% (2737/2972) of the CELF1-binding clusters are in genes (Fig. 3B). There are about twice as many intronic clusters than exonic clusters (1753 and 953 intronic and exonic clusters, respectively), which reveals an enrichment of exonic clusters when the relative lengths of introns and exons are taken into account. The exonic clusters are mainly located in the untranslated regions (UTR, Fig. 3B). We also investigated the type of genes with CELF1-binding clusters. A large majority of them (2564/2737) are protein-coding genes (Fig. 3C), yet a number of CELF1 binding clusters are located in lincRNA (139) or in antisense transcripts (39) (Fig. 3C).

Because CELF1 is known to preferentially associate with UGU repeats on RNAs, we next looked whether specific hexamers were overrepresented in our data sets using RSAT oligo-analysis [23]. As shown in Fig. 3D, TGT-containing hexamers are clearly favored in the DNA sequences identified in the CLIP-seq experiment, in agreement with CELF1 binding to UGU-rich motifs [8].

CELF1 is an evolutionary conserved RNA Binding protein. We therefore compared the overall overlap between its RNA targets identified by RIP-chip or CLIPseq in C2C12 cells ( [18], [22] respectively) and by RIP-chip in HeLa cells [24]. Supplementary Table 3 presents the full list of CELF1 targets and their presence in the tested datasets. As shown in Fig. 3E, About 20% (344/1232 + 344) of the CELF1 target genes identified by our CLIP-seq protocols are present in at least one list of previously published CELF1 targets identified in different cell types or using different techniques. A core set of 69 genes are identified in at least 2 other datasets and 4 genes (TBL1X, LNPEP, PRPF38B and TUBA1C) are common to all four datasets. If we take into account only the genes for which an exonic binding site is detected in our experiments (numbers in the parentheses), the percentage of CELF1 targets identified in at least one list of previously published CELF1 targets increases to about 33% (257/257 + 567).

### 3.3 CELF1 binding sites

**Fig. 4:**
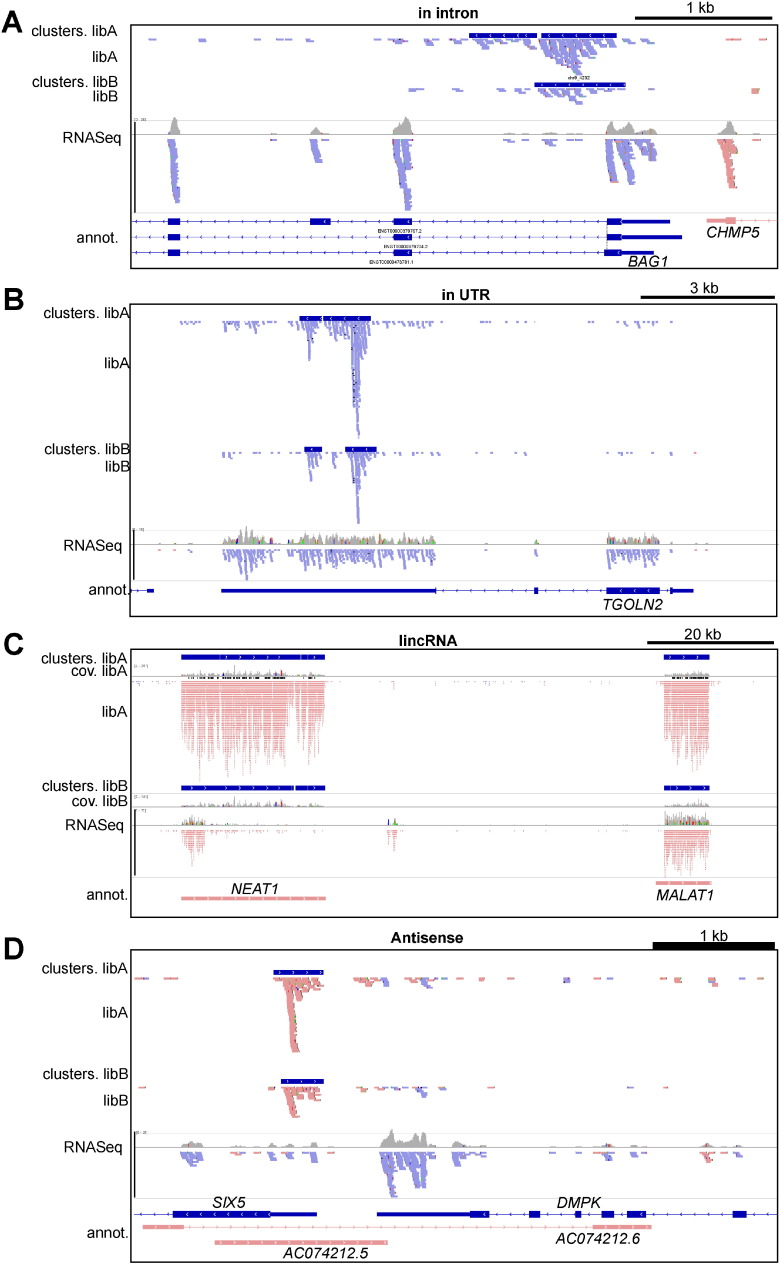
Examples of genes with CELF1 binding clusters. A) An intronic cluster in *BAG1* gene (a protein-coding gene). B) An exonic cluster in *TGOLN2* (a protein-coding gene) 3′UTR. C) Clusters in two lincRNAs, *NEAT1* and *MALAT1*/*NEAT2*. D) A cluster in an antisense RNA. Note that the *SIX5* and *DMPK* genes have the same orientation, and that the CLIP-seq cluster is in an antisense orientation relative to these two genes. In all panels, the sense reads are in red and the antisense reads in blue. The CELF binding clusters are in dark blue, with their orientations indicated by arrowheads.

Fig. 4 illustrates the different transcriptomic objects bound to CELF1 (intron, UTR, lincRNA and antisense RNA). CELF1 binds downstream to the first exon of the *BAG1* gene (Fig. 4A), which encodes an anti-apoptotic BCL2-associated protein involved in the maintenance of differentiating hematopoietic and neuronal cells [13]. This CELF1 binding sites is located close to an alternative 5′ splice site. Further experiments are needed to test if the interaction of CELF1 within *BAG1* intron 1 influences 5′ splice site selection, with a consequence on the sequence of the encoded protein and possibly its function. Similarly, we identified two CELF1 binding clusters upstream of the PKM exon 9 (Supplementary Table 1, Supplementary Table 2, and data not shown) in the HeLa cellular context where this exon is mainly skipped. As CELF1 over-expression force the usage of PKM exon 10 in C2C12 cells [11], it is tempting to postulate that CELF1 can directly regulate this splicing event in human Cells. However, this regulation of PKM splicing may be redundantly controlled by other RNA binding proteins as it was shown that the hnRNP proteins A1, A2 and PTB are critically involved in this process [4].

The binding of CELF1 in UTRs is illustrated in Fig. 4B**.** There**,** one libA cluster overlaps two libB clusters in the 3′UTR of the *TGOLN2* transcript. This interaction may impact the stability and/or translation of *TGOLN2* mRNA. CELF1 binding is also clearly identified on the two lincRNA *NEAT1* and *NEAT2*/*MALAT1* (Fig. 4C) that are localized to nuclear paraspeckles and SC35 nuclear subdomains [16]. There is no obvious hypothetical function for this interaction. While CELF1 might control lincRNA biology, the lincRNA might reciprocally serve as “CELF1 sponges”, as suggested for miRNA sponges [7], and hence regulate CELF1 function or availability. Finally, an example of CELF1-binding cluster in an antisense RNA is shown in Fig. 4D. The use of a strand-specific protocol for the CLIP-seq library preparation enables us to observe that the CELF1 protein binds to an RNA in the antisense orientation in between the *SIX5* and *DMPK* loci, and more precisely on the complementary strand of *SIX5* 5′UTR and *DMPK* 3′UTR.

This antisense RNA is absent from our mRNA-seq data, suggesting that it is a poorly or non-adenylated RNA, or a nuclear RNA remaining in an insoluble fraction during RNA extraction. Careful examination of ENCODE [9] RNASeq data (GSM765403/wgEncodeEH000172) shows that a transcript extending beyond the CELF1 binding sites and potentially overlapping the DMPK 3′UTR is present in the nucleus of HeLa S3 and other cells. This result is particularly interesting if we consider the involvement of CELF1 in myotonic dystrophy, type I (DM1). This genetic disease is caused by a CTG repeat expansion in the 3′UTR of the *DMPK* gene. The transcribed RNA containing the CUG repeat expansion causes formation of ribonuclear foci that sequester MBNL1 and up-regulates *CELF1* by incompletely understood mechanisms. The resulting imbalance between these two RNA-binding proteins is a major cause of DM1 symptoms [2]. It is therefore intriguing to find a CELF1-binding cluster in an antisense RNA very close to *DMPK* 3′UTR, but additional experiments are required to understand if it is a simple coincidence or if it has a significance for DM1 etiology.

### 3.4 Conclusions

CELF1 is a largely studied RNA-binding protein, probably due to its involvement in myotonic dystrophy, type I [2] and its relatively high abundance. CLIP-seq experiments have been carried out that aim at identifying in an unbiased manner the RNAs associated with CELF1 in mouse, including murine brains [6], murine skeletal muscle and heart [30] or the murine cell line C2C12 [22]. The present work complements these findings by identifying the RNA ligands of CELF1 in human cells. We provide a list of CELF1 binding clusters identified from two CLIP-seq libraries (Supplementary Table 1, Supplementary Table 2). The cross-validated clusters are high-confidence regions of interaction with CELF1, and the high proportion of libB clusters validated in libA demonstrates the specificity and reproducibility of the CLIP-seq libraries (see Fig. 2A). In addition, a large number of clusters identified in libA are not validated in libB, and we think that a main reason for this is the lower sequencing depths of libB. Accordingly, most non cross-validated libA clusters are probably bona fide CELF1-binding clusters. For example, the *LMO4* 3′UTR harbors a prominent CELF1 binding cluster in libA that is not identified as significant in libB. However, the reads in libA overlap an accumulation of reads in libB revealing CELF1 binding to *LMO4* 3′UTR, consistent with published literature (H.–H. [3]. Hence, our data provide a robust resource that will help characterizing genome-wide the functions of CELF1 in human.

## 4 Materials and methods

### 4.1 Cell extract, western blot and antibodies

HeLa-Kyoto Cells were grown at 5% CO2, 37 °C in DMEM (GIBCO BRL) complemented with 10% FCS, 100 U/ml penicillin and 100 μg/ml streptomycin (Invitrogen). Proteins were detected in western blots using anti-CELF1-2 (SantaCruz, 3B1, sc-20003), anti-CELF2 (1H2, sc-57731) and anti-ACTIN (Sigma-Aldrich, A5060).

### 4.2 CLIP-seq, mRNA-seq

We made CLIP experiments from untreated cells essentially as described [29] with some modifications in the library preparation. Briefly, we washed Hela-Kyoto cells twice with PBS (no Ca^2 +^, no Mg^2 +^), UV irradiated them 3 times at 4000 μJ/cm^2^ and 254 nm on ice, recovered them by scrapping and stored them at − 80 °C. We immunoprecipitated CELF1/RNA complexes from resuspended cell pellets as described [28], except that we incubated the cell lysates with 5 U RNase T1 for 10 min at 37 °C. We washed the beads 5 times with (Tris-HCl 50 mM, pH 7.4; NaCl 1 M; IGEPAL CA-630 0.5%; sodium deoxycholate 1%; SDS 0.1%; urea 2 M) and then 3 times with T4PNK + buffer (Tris-Cl 20 mM, pH 7.4; MgCl2 5 mM; IGEPAL CA-630 0.5%). We treated the beads for 35 min at 37 °C in T4 PNK + buffer in the presence of 40 UT4 PNK (Fermentas) and 50 μCi of ATP-gamma ^32^P to visualize RNA-protein complexes, or 1 mM unlabelled ATP to prepare the libraries. We eluted the RNA/protein complexes in NuPAGE 1 × loading buffer and fractionated them by PAGE in a neutral NuPAGE 4–12% bis-tris gel run in 1 × MOPS (Invitrogen). The complexes were transferred onto nitrocellulose membrane (protran BA-85) and the CELF1 complexes were cut out. RNAs were recovered following proteinase K digestion in PK buffer (Tris-HCl 100 mM, pH 7.4; NaCl 50 mM; EDTA 10 mM; proteinase K (Sigma) 2 mg/ml) for 30 min at 37 °C. The digestion was pursued in PK buffer with urea (7 M) for 30 min at 60 °C. Proteins were then extracted by phenol/CHCl_3_ and RNA precipitated with ammonium acetate and isopropanol using glycogen as carrier. RNAs were washed twice with 80% ethanol, dried and resuspended in water. For cloning and sequencing, radioactive experiments were run in parallel with nonradiolabeled experiments and nonradioactive samples were further used for cloning and sequencing. The RNA fragments were ligated to adapters, reverse transcribed and amplified by PCR following manufacturer instruction using the Small RNA expression kit (Ambion # 4397682).

For mRNA-seq, total RNAs were extracted from growing Hela-Kyoto cells using RNAeasy columns (Qiagen). Poly(A) + RNAs were selected on oligo-dT (Promega) and partially fragmented using RNAse T1. The size of the fragments was controlled using a bioanalyzer. We constructed a cDNA library for deep sequencing following manufacturer instructions using the Small RNA expression kit as described above. One RNASeq library was prepared and sequenced.

SOLiD sequencing was performed at the Genoscope on a SoliD 3 system. Two different CLIP-seq libraries and one mRNA-seq library were sequenced for 50 cycles following manufacturer recommendations.

### 4.3 Sequence data analysis

The detailed procedure and the R script are available as supplementary materials.

#### 4.3.1 Filtering and trimming

Raw sequencing data were collected as colorfasta (.csfasta) and quality files (.qual). Sequence data were trimmed with cutadapt [21] to remove SoliD adapters (CGCCTTGGCCGTACAGCA); reads kept were requested to have a minimal size of 30 nucleotides. Based on sequence quality analysis, we discarded any sequences harboring an uncalled base in the first 29 nucleotides.

#### 4.3.2 Mapping to hg19

The resulting sequence files were mapped with SHRIMP 2 [25] to the human genome (hg19) after converting the genome to colorspace. The resulting bam files were merged by library to obtain the 2 CLIPSeq datasets, libA and libB and the RNASeq dataset libC. After mapping, in each library reads considered as duplicated based on the flag, chr, start and CIGAR fields were removed to keep only one read.

#### 4.3.3 Identification of enriched clusters

Read cluster identification was conducted in parallel on the two libraries using FindPeaks 4.0 [10] using the RNASeq as a comparison file. Only reads enriched in the CLIPSeq libraries were kept for further analysis.

#### 4.3.4 Annotation of enriched clusters

Cluster strand was defined based on mapped read strand. The annotation of the cluster based on their genomic location was conducted using R script and the GENCODE annotation (Release 19 (GRCh37.p13)).

#### 4.3.5 Comparison with previously identified CELF1 target

Binding data for CELF1 targets were obtained from CLIPSeq and RIP-Chip experiments published in [18], [22], [24] and gene identifiers were converted to human Ensembl gene ID for comparison purpose.

The following are the supplementary data related to this article.

Supplementary Table 1 CELF1-binding clusters library A. Supplementary Table 1

Supplementary Table 2 CELF1-binding clusters library B. Supplementary Table 2

Supplementary Table 3 CELF1 conserved targets. Supplementary Table 3

Detailed_procedure. Image 1

Solid reads quality analysis script. Image 2

FindPeaks analysis script. Image 3
